# Nonlinear relationship between estimated pulse wave velocity and chronic kidney disease: analyses of NHANES 1999–2020

**DOI:** 10.3389/fmed.2025.1560272

**Published:** 2025-06-12

**Authors:** Wenshi Li, Rui Xu, Honghao Yan, Zhan Li, Jiale Sun, Lin Wang

**Affiliations:** ^1^Department of Nephrology, Longhua Hospital, Shanghai University of Traditional Chinese Medicine, Shanghai, China; ^2^School of Basic Medical Sciences, Zhejiang Chinese Medical University, Hangzhou, China

**Keywords:** NHANES, estimated pulse wave velocity, chronic kidney disease, RCS, arterial stiffness

## Abstract

**Background:**

Limited data on the correlation between estimated pulse wave velocity (ePWV) and chronic kidney disease (CKD) necessitate further investigation. This study aims to explore the association between ePWV and the prevalence of CKD.

**Methods:**

A cross-sectional study was conducted with 41,411 participants from the 1999–2020 National Health and Nutrition Examination Survey (NHANES). ePWV was calculated using an established equation from the Reference Values for Arterial Stiffness Collaboration, incorporating age and mean blood pressure. CKD prevalence was assessed as the primary outcome. Weighted logistic regression and linear models were applied for statistical analysis, with Restricted Cubic Splines (RCS) used to evaluate potential nonlinear associations. Subgroup analyses were conducted to assess variations and ensure the robustness of results.

**Results:**

Higher ePWV was consistently associated with an increased prevalence of CKD. RCS analysis identified a significant positive nonlinear relationship. Subgroup analyses revealed sex-based and glucose metabolism abnormality-based differences, highlighting interactive correlations that provided further insights into the ePWV-CKD relationship.

**Conclusion:**

This study demonstrates a strong positive association between ePWV and CKD prevalence, underscoring the importance of monitoring arterial stiffness. The use of RCS and subgroup analyses enriched the findings and offered valuable directions for future research.

## 1 Introduction

Chronic kidney disease (CKD) is defined by persistent abnormalities in kidney structure or function lasting for more than 3 months (1). CKD is a progressive condition associated with significant morbidity and mortality and represents a critical public health challenge, affecting approximately 8–16% of the global population (2). By 2040, CKD is projected to become the fifth leading cause of death worldwide (3). Alarmingly, fewer than 10% of individuals with CKD are aware of their diagnosis, both in developed and developing nations (4). Early identification of individuals at higher risk of CKD is therefore essential for timely intervention and improved outcomes.

The kidneys receive the highest blood flow per gram of tissue among all organs, supported by their unique microvascular structures, including the glomerular, peritubular, and renal medullary microcirculations (5). Fluctuations in renal blood flow significantly affect key renal functions, such as glomerular filtration, tubular reabsorption, and blood pressure regulation. Consequently, vascular health, particularly arteriosclerosis, has garnered increasing attention in the study of kidney diseases. Recent evidence indicates that heightened aortic stiffness exacerbates pressure and flow pulsatility, transmitting excessive pulsatile energy to the peripheral vasculature (6). This mechanism is particularly relevant to kidney injury, given the renal microvasculature’s unique susceptibility due to continuous passive renal perfusion, low input impedance, and diminished wave reflections. These features render the kidneys particularly vulnerable to pulsatile energy transfer, which contributes to glomerulosclerosis and the progressive deterioration of renal function (7, 8). Prospective observational studies have consistently demonstrated a strong association between increased arterial stiffness and reduced estimated glomerular filtration rate (eGFR), albuminuria, and an elevated risk of CKD (6, 9–22). However, there remains ongoing debate regarding the reliability of arterial stiffness as a predictor of CKD onset and progression, potentially due to the variability in arterial stiffness markers employed across studies (9, 12, 16–22).

Aortic pulse wave velocity (PWV) is widely regarded as the non-invasive gold standard for assessing arterial stiffness, with carotid-femoral pulse wave velocity (cfPWV) being the most extensively validated and reliable indicator of aortic stiffness (23–25). Despite standardized protocols for measuring PWV and cfPWV (26), their assessment requires costly, specialized equipment, limiting their application in routine clinical practice, particularly in primary care settings. To address these limitations, estimated pulse wave velocity (ePWV) has emerged as a more accessible alternative, demonstrating predictive accuracy comparable to cfPWV. ePWV can be easily derived using age and mean arterial pressure (MAP) based on formulas developed by the Reference Values for Arterial Stiffness Collaboration (27, 28). Notably, ePWV has been shown to independently predict composite cardiovascular outcomes, outperforming traditional risk scores such as the Systemic Coronary Risk Evaluation (SCORE) and the Framingham Risk Score (FRS), as well as cfPWV. For example, the MORGAM Prospective Cohort Project demonstrated that ePWV predicts all-cause mortality (ACM) independent of standard cardiovascular risk factors, highlighting its role as more than just a cardiovascular risk marker (29). Recent studies further support the utility of ePWV as a predictor of CKD. For instance, a study involving 4,838 participants from the Vitamin D Assessment (ViDA) cohort, with a follow-up of 10.5 years, found that higher ePWV was associated with an increased risk of CKD development, even in participants without prior CKD (21). Building on this evidence, the present study aims to investigate the association between ePWV and CKD prevalence using a comprehensive dataset from the National Health and Nutrition Examination Survey (NHANES) covering 1999–2020. By leveraging a large, representative population sample, this study seeks to provide findings with broad generalizability.

In conclusion, we hypothesize that there is a positive association between ePWV levels and CKD prevalence. The results of this study may offer a practical and reliable approach to CKD risk assessment, contributing to the development of innovative strategies for early clinical intervention and improved disease management.

## 2 Methods

### 2.1 Study design and population

The National Health and Nutrition Examination Survey (NHANES) is an ongoing annual survey conducted by the National Center for Health Statistics (NCHS), a division of the Centers for Disease Control and Prevention (CDC) in the United States. NHANES uses a stratified, multistage probability sampling design to select households nationwide. Within selected households, a subset of adults is randomly chosen to participate. Data are collected on demographics, lifestyle factors, and health outcomes through standardized interviews, physical examinations, and laboratory tests. All participants provided informed consent in compliance with the Declaration of Helsinki, and the study protocol was approved by the NCHS Research Ethics Committee. Detailed information about NHANES is publicly available on the official website^1^.

**Figure 1:**
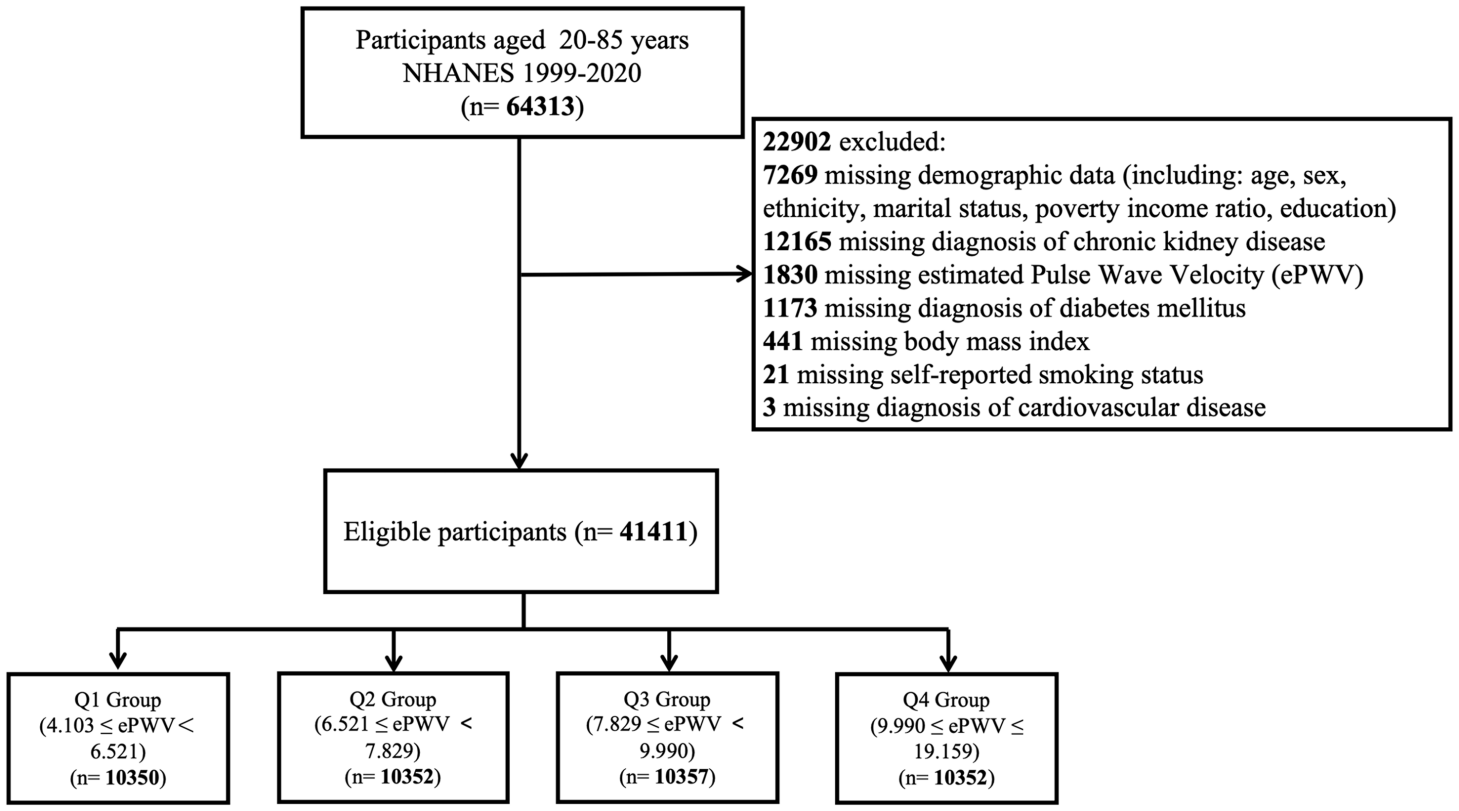
Flowchart showing the selection of the studied population.

Data for this study were obtained from NHANES cycles spanning 1999–2020. Among a total of 64,313 participants aged 20–85 years, 22,902 individuals were excluded due to missing data. As a result, 41,411 participants were included in the final analysis. The selection process is illustrated in Figure 1.

### 2.2 Diagnosis of CKD

CKD was defined using the KDIGO 2021 guidelines. Briefly, data on urinary albumin-to-creatinine ratios (ACRs) and estimated glomerular filtration rate (eGFR) were extracted from NHANES. ACR categories were classified as <30 mg/g (A1), 30–300 mg/g (A2), and >300 mg/g (A3). eGFR was calculated using the Chronic Kidney Disease Epidemiology Collaboration (CKD-EPI) equation and classified into the following stages: G1 (≥90 mL/min/1.73 m^2^), G2 (60–89 mL/min/1.73 m^2^), G3a (45–59 mL/min/1.73 m^2^), G3b (30–44 mL/min/1.73 m^2^), G4 (15–29 mL/min/1.73 m^2^), and G5 (<15 mL/min/1.73 m^2^). CKD was diagnosed in participants with eGFR <60 mL/min/1.73 m^2^ or ACR > 30 mg/g.

CKD patients were further stratified into three prognostic risk categories based on their likelihood of disease progression: moderate risk (G3a and A1, or G1–G2 and A2), high risk (G3b and A1, G3a and A2, or G1–G2 and A3), and very high risk (G4–G5, G3b and A2–A3, or G3a and A3) (KDIGO 2021 Clinical Practice Guidelines for Glomerular Diseases).

### 2.3 Calculation of ePWV

The Equation 1 derived from the Reference Values for Arterial Stiffness Collaboration was used to calculate ePWV. According to the Equation 1, ePWV is calculated from age and mean blood pressure (MBP):


(1) $$\begin{array}{l} 9.587-0.402\times \text{age}+4.560\times 10^{-3}\times {\text{age}}^{2}\\ -2.621\times 10^{-5}\times {\text{age}}^{2}\times \text{MBP}+3.176\times 10^{-3}\\ \times \text{age}\times \text{MBP}-1.832\times 10^{-2}\times \text{MBP} \end{array}$$

MBP is calculated as diastolic blood pressure + 0.4 × (systolic blood pressure - diastolic blood pressure).

### 2.4 Covariates

Potential confounders influencing the association between ePWV and CKD were accounted for in multivariable adjustment models. Covariates included baseline demographic data and physical examination measurements, such as age (years), sex (male, female), ethnicity (Non-Hispanic White, Non-Hispanic Black, Mexican American, Other Hispanic, Other race, including multiracial), marital status (married, living with a partner, separated, divorced, widowed, or never married), educational attainment (<9th grade, 9–11th grade, high school graduate, some college or associate degree, college graduate or higher), smoking status (never, former, or current), income level (high, middle, or low), hypertension (yes/no), cardiovascular disease (yes/no), diabetes mellitus (yes, impaired fasting glucose, impaired glucose tolerance, or no), CKD stages (by ACR and eGFR), and CKD prognosis (low, moderate, high, or very high risk). These data were obtained through participant self-reports. Detailed measurement techniques are available on the NHANES website (see text footnote 1).

### 2.5 Statistical analysis

Descriptive analyses were conducted to compare categorical and continuous variables. Chi-squared (*χ*^2^) tests were used for categorical variables, while continuous variables were analyzed using Student’s t-tests and analysis of variance (ANOVA) for normally distributed data. Categorical variables were expressed as numbers and weighted proportions, and continuous variables were presented as weighted means with standard errors.

Three binary logistic regression models were constructed to estimate odds ratios (ORs) and 95% confidence intervals (CIs) for the association between ePWV and CKD:

Model 1: Unadjusted.

Model 2: Adjusted for age (continuous), sex, ethnicity, marital status, poverty-income ratio, and educational attainment.

Model 3: Further adjusted for the body mass index, smoke status, cardiovascular disease, diabetes mellitus.

Restricted cubic spline (RCS) curves were applied to evaluate potential nonlinear relationships between ePWV and CKD. Multivariable-adjusted ORs (red solid line) with 95% CIs (pink shaded area) were plotted, adjusting for all covariates included in Model 3. Subgroup analyses were performed using Model 3 to examine potential effect modifications across stratified populations. Each subgroup analysis controlled for all covariates in Model 3, except the stratification variable. All statistical analyses were conducted using R software (version 4.3.3). A two-tailed *p*-value <0.05 was considered statistically significant.

## 3 Results

### 3.1 Characteristics of study participants

A total of 41,411 participants were included in this study, with a mean age of 46.91 ± 0.21 years. Of these, 50.77% were male, and 49.23% were female. The weighted baseline characteristics of the participants are summarized in Table 1. The mean estimated pulse wave velocity (ePWV) was 8.08 ± 0.02 m/s. Participants were categorized into quartiles based on ePWV values: 4.103–6.521 (Q1), 6.521–7.829 (Q2), 7.829–9.990 (Q3), and 9.990–19.159 (Q4), with Q1 serving as the reference group (Figure 1). The overall prevalence of chronic kidney disease (CKD) among participants was 13.82%.

**Table 1:** Characteristics of study participants^a^.

Characteristic	Overall	ePWVQ1	ePWVQ2	ePWVQ3	ePWVQ4	*p* value
	*n* = 41,411 (%)	*n* = 10,350 (%)	*n* = 10,352 (%)	*n* = 10,357 (%)	*n* = 10,352 (%)	
Age	46.91 (0.21)	29.93 (0.13)	40.33 (0.19)	55.17 (0.14)	71.18 (0.15)	< 0.0001
ePWV (m/s)	8.08 (0.02)	6.10 (0.00)	7.10 (0.01)	8.78 (0.01)	11.61 (0.02)	< 0.0001
BMI (kg/m2)	28.90 (0.07)	26.89 (0.10)	29.69 (0.12)	30.19 (0.12)	28.88 (0.09)	< 0.0001
Sex						< 0.0001
Female	20,709 (50.77)	6,046 (57.67)	4,655 (44.32)	4,971 (48.45)	5,037 (53.64)	
Male	20,702 (49.23)	4,304 (42.33)	5,697 (55.68)	5,386 (51.55)	5,315 (46.36)	
Race/ethnicity						< 0.0001
Mexican American	6,998 (8.07)	2,130 (11.91)	1815 (9.06)	1721 (5.87)	1,332 (3.81)	
Non-Hispanic Black	8,447 (10.49)	1978 (11.04)	2,128 (10.74)	2,376 (10.63)	1965 (9.07)	
Non-Hispanic White	18,842 (69.19)	4,159 (61.58)	4,502 (67.06)	4,429 (72.79)	5,752 (78.94)	
Other Hispanic	3,303 (5.10)	962 (7.21)	788 (5.13)	902 (4.24)	651 (3.07)	
Other race - including multi-racial	3,821 (7.15)	1,121 (8.27)	1,119 (8.01)	929 (6.47)	652 (5.11)	
Marital status						< 0.0001
Married	21,539 (55.31)	4,102 (41.16)	5,599 (58.36)	6,180 (64.87)	5,658 (58.37)	
Living with partner	2,988 (7.65)	1,363 (13.39)	922 (8.39)	495 (4.60)	208 (2.13)	
Separated	1,322 (2.32)	289 (2.17)	379 (2.74)	427 (2.71)	227 (1.31)	
Divorced	4,296 (10.17)	488 (4.73)	1,012 (10.25)	1,599 (14.93)	1,197 (11.49)	
Widowed	3,241 (5.46)	37 (0.31)	143 (1.22)	565 (3.82)	2,496 (22.31)	
Never married	8,025 (19.09)	4,071 (38.24)	2,297 (19.03)	1,091 (9.07)	566 (4.38)	
Education status						< 0.0001
College graduate or above	9,197 (29.17)	2,459 (29.69)	2,545 (31.30)	2,297 (29.25)	1896 (24.92)	
Some college or AA degree	12,148 (31.78)	3,398 (33.13)	3,287 (33.03)	2,977 (31.75)	2,486 (27.81)	
High school graduate	9,620 (23.68)	2,356 (22.72)	2,314 (22.10)	2,447 (24.60)	2,503 (26.28)	
9–11th grade	5,885 (10.19)	1,479 (10.65)	1,418 (9.55)	1,424 (9.38)	1,564 (11.63)	
Less than 9th grade	4,561 (5.19)	658 (3.80)	788 (4.03)	1,212 (5.03)	1903 (9.36)	
Smoke status						< 0.0001
Never	22,354 (54.27)	6,373 (59.80)	5,825 (55.75)	5,111 (50.43)	5,045 (49.07)	
Former	10,264 (24.84)	1,292 (14.31)	1843 (20.25)	2,959 (29.66)	4,170 (41.12)	
Current	8,793 (20.89)	2,685 (25.90)	2,684 (24.00)	2,287 (19.91)	1,137 (9.80)	
Income level						< 0.0001
High income	13,041 (43.28)	2,835 (35.55)	3,537 (46.33)	3,935 (52.48)	2,734 (37.04)	
Middle income	15,750 (35.89)	3,895 (37.49)	3,710 (33.64)	3,583 (31.20)	4,562 (43.74)	
Low income	12,620 (20.83)	3,620 (26.96)	3,105 (20.03)	2,839 (16.33)	3,056 (19.22)	
CKD						< 0.0001
No	34,056 (86.18)	9,809 (94.93)	9,528 (93.33)	8,645 (86.18)	6,074 (61.70)	
Yes	7,355 (13.82)	541 (5.07)	824 (6.67)	1712 (13.82)	4,278 (38.30)	
CKD prognosis						< 0.0001
Low risk	34,056 (86.18)	9,809 (94.93)	9,528 (93.33)	8,645 (86.18)	6,074 (61.70)	
Moderate risk	5,091 (10.14)	478 (4.52)	696 (5.81)	1,268 (10.62)	2,649 (24.79)	
High risk	1,427 (2.46)	51 (0.48)	95 (0.70)	285 (2.20)	996 (8.62)	
Very high risk	837 (1.21)	12 (0.07)	33 (0.17)	159 (1.00)	633 (4.89)	
CKD stages by ACR						< 0.0001
A1	36,398 (90.80)	9,835 (95.13)	9,615 (94.22)	9,053 (90.18)	7,895 (79.75)	
A2	4,166 (7.87)	463 (4.44)	635 (5.13)	1,060 (8.15)	2008 (16.99)	
A3	847 (1.33)	52 (0.43)	102 (0.65)	244 (1.67)	449 (3.25)	
CKD stages by eGFR						< 0.0001
G1	24,484 (60.53)	9,247 (87.27)	7,934 (72.97)	5,439 (48.99)	1864 (16.91)	
G2	13,370 (33.01)	1,064 (12.41)	2,292 (25.89)	4,303 (45.50)	5,711 (57.59)	
G3a	2,363 (4.57)	28 (0.25)	94 (0.97)	426 (4.30)	1815 (17.14)	
G3b	859 (1.41)	4 (0.02)	14 (0.12)	120 (0.75)	721 (6.47)	
G5	92 (0.12)	1 (0.00)	12 (0.04)	29 (0.22)	50 (0.27)	
G4	243 (0.37)	6 (0.05)	6 (0.02)	40 (0.24)	191 (1.62)	
Hypertension						< 0.0001
No	24,042 (62.88)	9,644 (93.03)	7,693 (74.63)	4,506 (46.17)	2,199 (22.61)	
Yes	17,369 (37.12)	706 (6.97)	2,659 (25.37)	5,851 (53.83)	8,153 (77.39)	
CVD						< 0.0001
No	36,969 (91.59)	10,194 (98.67)	9,960 (96.63)	9,117 (89.89)	7,698 (75.35)	
Yes	4,442 (8.41)	156 (1.33)	392 (3.37)	1,240 (10.11)	2,654 (24.65)	
DM						< 0.0001
DM	7,044 (13.14)	307 (2.54)	1,094 (8.96)	2,552 (19.57)	3,091 (26.55)	
IFG	1877 (4.66)	197 (2.12)	427 (4.14)	605 (6.21)	648 (7.08)	
IGT	1,155 (2.95)	161 (1.63)	297 (3.09)	301 (3.25)	396 (4.31)	
No	31,335 (79.26)	9,685 (93.71)	8,534 (83.81)	6,899 (70.97)	6,217 (62.06)	

Data are expressed as number (weighted proportions) for categorical variables and as weighted means (standard error) for continuous variables. ACR, urinary albumin-to-creatinine ratio; BMI, body mass index; CKD, chronic kidney disease; CVD, cardiovascular disease; DM, diabetes mellitus; eGFR, estimated glomerular filtration rate; ePWV, estimated Pulse Wave Velocity; IFG, impaired fasting glucose; IGT, impaired glucose tolerance.

a Two-sided *p* values show results of univariate comparisons among four groups according to the quartile of ePWV. All categorical variables were tested with the *χ*^2^ test.

Compared to those in Q1, individuals in Q4 were more likely to be older, non-Hispanic White, married, have lower educational attainment, and exhibit a higher prevalence of hypertension, cardiovascular disease, and diabetes mellitus.

### 3.2 Association between ePWV and CKD

Binary logistic regression analysis identified ePWV as a significant risk factor for CKD (Table 2). After adjusting for all potential confounders, each unit increase in ePWV was associated with a 50% higher risk of CKD (OR = 1.50; 95% CI: 1.47–1.54; *p* < 0.0001). This finding highlights a strong positive association between elevated ePWV levels and CKD.

**Table 2:** Crude and adjusted association between chronic kidney disease with estimated Pulse Wave Velocity Values are expressed as OR (95% CI).

Model		Model 1 (OR)	*p* values	Model 2 (OR)	*p* values	Model 3 (OR)	*p* values
ePWV (m/s) (Continuous)	Per 1 m/s Increase	1.58 (1.55, 1.61)	<0.0001	1.58 (1.54, 1.61)	<0.0001	1.50 (1.47, 1.54)	<0.0001
ePWV levels	Q1 [4.103, 6.521)	1.00 (Reference)		1.00 (Reference)		1.00 (Reference)	
	Q2 [6.521, 7.829)	1.34 (1.15, 1.56)	<0.001	1.46 (1.25, 1.71)	<0.0001	1.26 (1.08, 1.48)	0.003
	Q3 [7.829, 9.990)	3.00 (2.60, 3.48)	<0.0001	3.33 (2.84, 3.90)	<0.0001	2.39 (2.03, 2.80)	<0.0001
	Q4 [9.990, 19.159)	11.63 (10.22, 13.22)	<0.0001	11.44 (9.80, 13.35)	<0.0001	7.61 (6.49, 8.92)	<0.0001
	*p* for trend		<0.0001		<0.0001		<0.0001

Model 1: unadjusted model.

Model 2: adjusted for age (continuous), sex (Male, Female), ethnicity (Non-Hispanic White, Non-Hispanic Black, Other Hispanic, Mexican American, Other Race - Including Multi-Racial), marital status (living with a spouse / partner, or living without a spouse / partner), poverty income ratio, and educational level (divided into less than 9th grade, 9–11th grade, high school graduate, some college or AA degree, college graduate or above).

Model 3: Further adjusted for the body mass index, smoke status, cardiovascular disease, diabetes mellitus.

CI, confidence interval; ePWV, estimated Pulse Wave Velocity; OR, odds ratio.

In the fully adjusted model, participants in Q4 demonstrated a significantly higher likelihood of CKD compared to those in Q1 (OR = 7.61; 95% CI: 6.49–8.92; *p* < 0.0001). Additionally, a significant trend was observed across ePWV quartiles (*p* for trend < 0.0001).

### 3.3 Nonlinear relationship between ePWV and CKD

**Figure 2:**
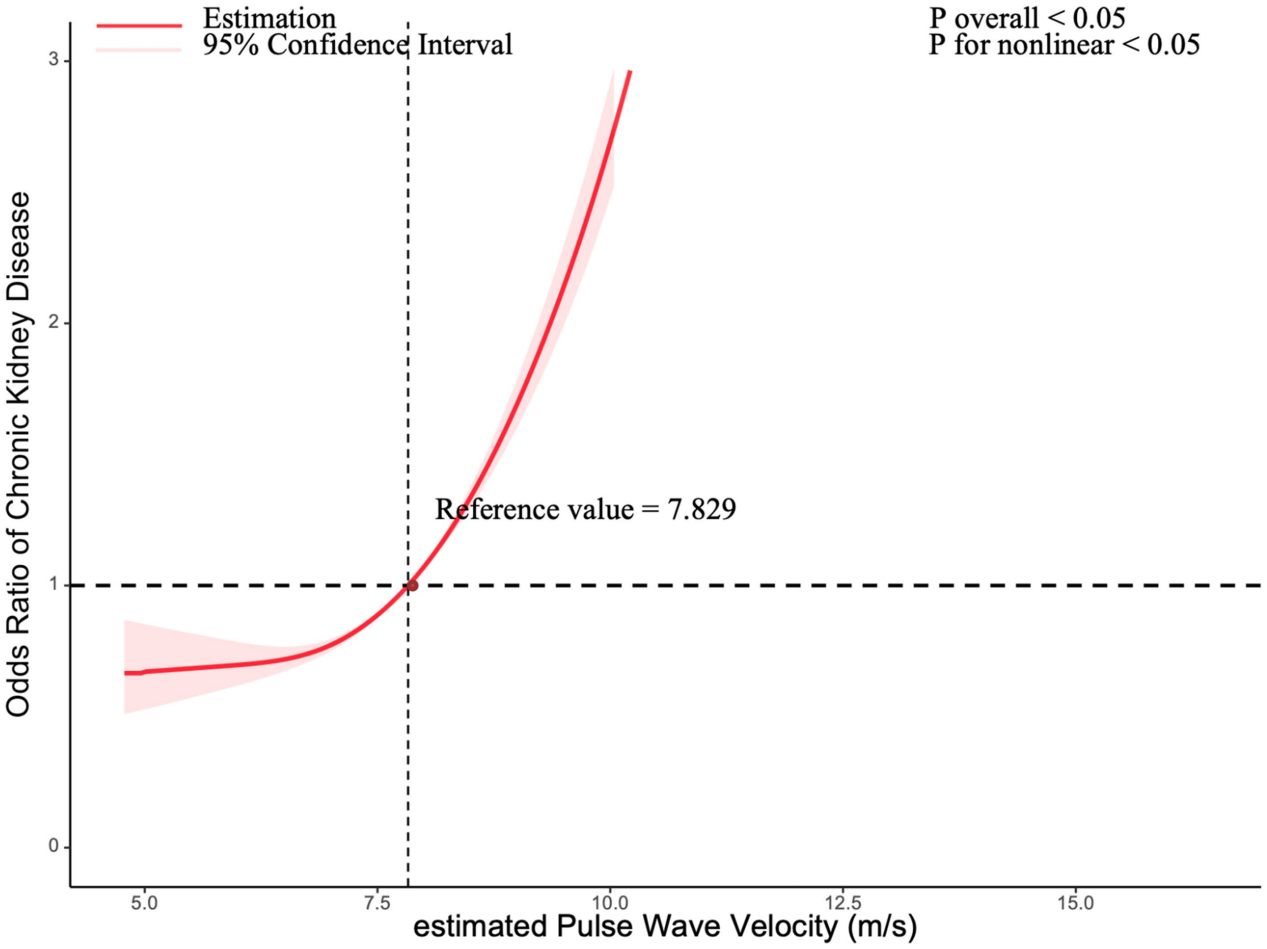
Association between chronic kidney disease with estimated pulse Wave Velocity in a restricted cubic spline model. Multivariable adjusted odds ratios (red solid line) with 95% confidence interval (pink shaded area) for the association between chronic kidney disease with estimated Pulse Wave Velocity. Adjusted for sex (Male, Female), ethnicity (Non-Hispanic White, Non-Hispanic Black, Other Hispanic, Mexican American, Other Race - Including Multi-Racial), marital status (living with a spouse / partner, or living without a spouse / partner), poverty income ratio, educational level (divided into less than 9th grade, 9–11th grade, high school graduate, some college or AA degree, college graduate or above), body mass index, smoke status, cardiovascular disease, and diabetes mellitus.

A restricted cubic spline (RCS) model was used to investigate the nonlinear relationship between ePWV and CKD among all participants. After adjusting for confounders, the RCS model revealed a significant nonlinear dose–response relationship (*p* < 0.05 for both significance and nonlinearity). When ePWV exceeded 7.829 m/s, the relative risk of CKD increased, with odds ratios (ORs) surpassing 1. The results are visualized in Figure 2.

### 3.4 Subgroup analysis

Subgroup analyses were conducted to evaluate potential differences in the association between ePWV and CKD across various population subgroups. Stratified analyses revealed that older participants, males, individuals without diabetes mellitus, and those without hypertension were more sensitive to changes in ePWV. Significant differences were observed among groups stratified by sex and glucose metabolism abnormalities (*p* < 0.0001 for both).

**Figure 3:**
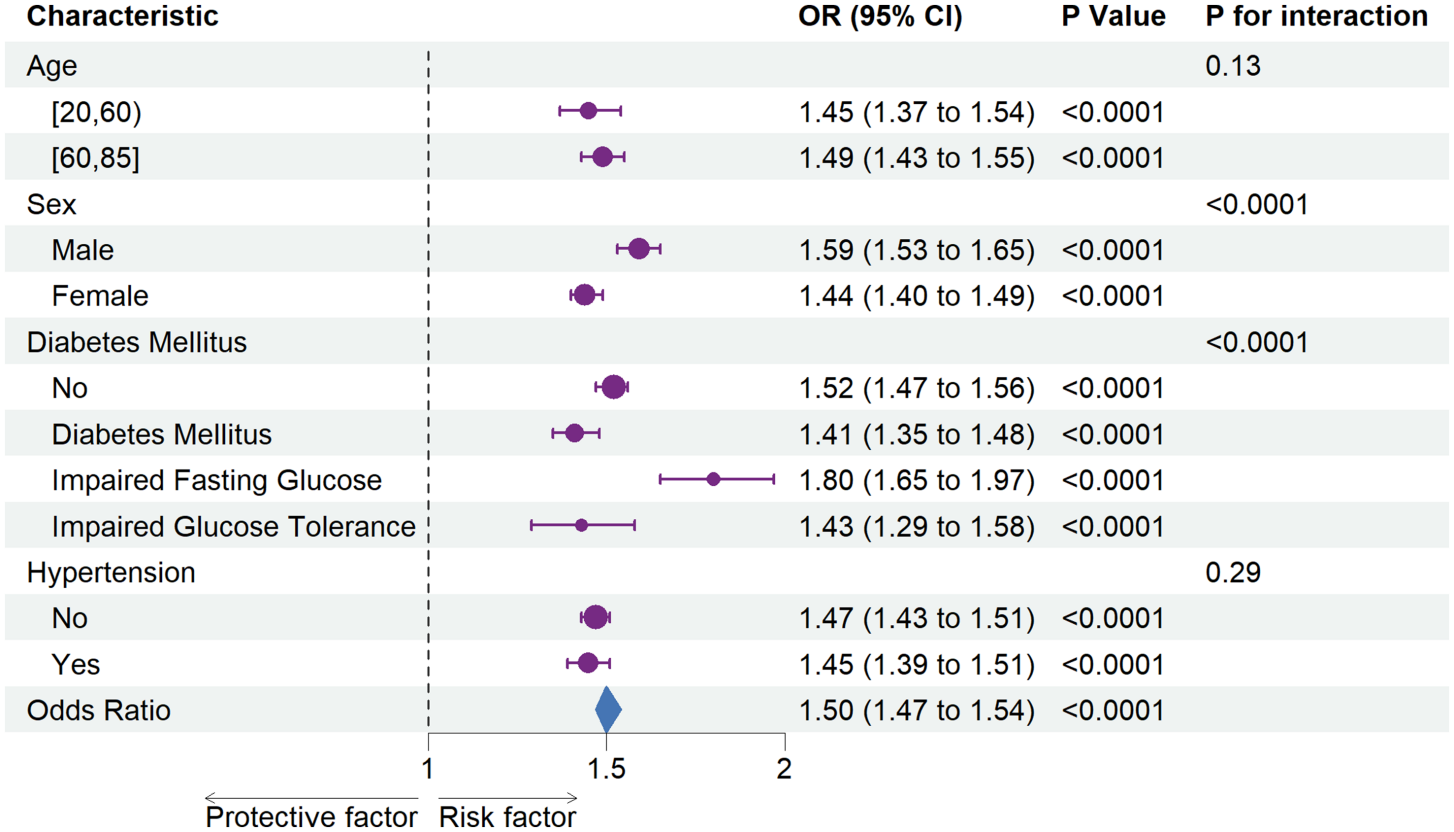
The association between chronic kidney disease with estimated pulse Wave Velocity in subgroups Each stratification was adjusted for sex (Male, Female), ethnicity (Non-Hispanic White, Non-Hispanic Black, Other Hispanic, Mexican American, Other Race - Including Multi-Racial), marital status (living with a spouse / partner, or living without a spouse / partner), poverty income ratio, educational level (divided into less than 9th grade, 9–11th grade, high school graduate, some college or AA degree, college graduate or above), body mass index, smoke status, cardiovascular disease, and diabetes mellitus, except the stratification factor itself. OR, odds ratio; CI, confidence interval.

In the overall analysis, the combined OR was 1.50 (95% CI: 1.47–1.54; *p* < 0.0001), indicating that each unit increase in ePWV was associated with a 50% increase in CKD incidence. These findings are illustrated in Figure 3.

## 4 Discussion

To the best of our knowledge, this is the first study to evaluate the association between estimated pulse wave velocity (ePWV) and chronic kidney disease (CKD) using NHANES data spanning 1999 to 2020. Our findings demonstrate a non-linear positive correlation between ePWV and CKD prevalence. Additionally, we observed that as ePWV quartiles increased, the risk of CKD rose proportionally. These results suggest that ePWV, a simple surrogate for aortic stiffness, could serve as a valuable risk indicator to improve CKD identification in primary care settings.

In 2020, the Global Burden of Disease Group identified CKD as one of the top 10 adverse prognostic factors worldwide. Among CKD-related complications, cardiovascular disease (CVD) is the leading cause of mortality (30, 31). Emerging research highlights that arteriosclerosis, historically considered an independent risk factor for CVD, also contributes to CKD onset and progression (32). Arteriosclerosis increases arterial rigidity, amplifying pressure wave reflection and oscillations in blood flow. These effects are particularly pronounced in the kidneys, which are high-flow, low-impedance end organs. Such amplified hemodynamic forces damage the renal microvasculature, impairing glomerular filtration rate (GFR) and reducing renal function (6, 33). Furthermore, arteriosclerosis triggers microvascular remodeling in the kidneys, including an increased media-to-lumen ratio, which raises peripheral resistance and diminishes renal autoregulatory capacity. This remodeling, coupled with arterial stiffness, leads to cortical artery loss and vascular damage, further reducing GFR and impairing renal function (34). This remodeling, coupled with arterial stiffness, leads to cortical artery loss and vascular damage, further reducing GFR and impairing renal function (6). Imaging studies corroborate these findings, demonstrating that aortic stiffening and excessive flow pulsatility cause microvascular injury and functional decline in the kidneys (23).

Arterial stiffness, often quantified by pulse wave velocity (PWV), is widely recognized as a key biomarker of vascular health. The carotid-femoral PWV (cfPWV) is considered the gold standard for assessing aortic stiffness, as recommended by the European Society of Hypertension/European Society of Cardiology (ESH/ESC) and the American Heart Association (AHA) guidelines (5, 24, 35). However, evidence on the association between PWV and CKD has been inconsistent. For instance, a prospective cohort of 7,154 Chinese adults without baseline CKD found that higher cfPWV (≥16.7 m/s) was significantly associated with increased CKD risk over 3 years (20). Similarly, other studies, including the AGES-Reykjavik study and the Rotterdam Study, demonstrated that elevated cfPWV predicted GFR decline and renal function deterioration over follow-up periods of 5.3 and 11 years, respectively, though no association was observed with albuminuria (6, 11, 14).

Conversely, Madero et al. found that aortic PWV was associated with CKD incidence but not with rapid kidney function decline in a cohort of 2,129 older adults over 8.9 years (12). Similarly, the Framingham Heart Study linked cfPWV to albuminuria but not to mild-to-moderate CKD (16).

Alternative methods, such as brachial-ankle PWV (baPWV), are commonly used in Asia but demonstrate inconsistent correlations with CKD. For instance, a Japanese study found that for every 1 m/s increase in baPWV, the likelihood of GFR decline increased by 36% (*p* < 0.01) (17), whereas another study in 913 CKD patients found no significant association between baPWV and GFR decline (22). Additionally, baPWV is poorly correlated with cfPWV, limiting its utility as a surrogate for aortic stiffness (36).

Despite its clinical relevance, aortic stiffness has not been widely adopted in routine practice, largely due to technological and logistical challenges. For example, cfPWV measurement requires specialized equipment and expertise, limiting its accessibility. In contrast, ePWV offers a simple, cost-effective alternative with comparable predictive value. Derived from age and mean arterial pressure (MAP), ePWV requires no specialized equipment and can be easily implemented in clinical settings (27, 28). Recent studies, including one based on the Vitamin D Assessment (ViDA) cohort, support the utility of ePWV as a predictor of CKD risk (21).

Our study confirms these findings. Logistic regression analysis revealed that each 1 m/s increase in ePWV was associated with a 50% higher risk of CKD (OR = 1.50; 95% CI: 1.47–1.54; *p* < 0.0001). A restricted cubic spline (RCS) model further demonstrated a notable rise in CKD prevalence when ePWV exceeded 7.829 m/s. These results suggest that ePWV may be a risk factor for CKD and a valuable biomarker for monitoring CKD progression.

Subgroup analyses revealed that the association between ePWV and CKD was consistent across age and hypertension subgroups, suggesting that arteriosclerosis impacts CKD prevalence independently of blood pressure levels. This suggests that even in patients with well-controlled blood pressure, those with CKD should remain vigilant regarding the risk of arterial stiffness. Interestingly, significant interactions were observed between ePWV and sex, as well as glycemic status. Male participants exhibited higher susceptibility to ePWV-related CKD risk (OR = 1.59 in men vs. OR = 1.44 in women; *p* for interaction < 0.0001), consistent with prior research showing that men have higher PWV levels than women (37). Stratification by glycemic status suggested potential synergistic effects between elevated blood glucose and arterial stiffness on CKD risk, warranting further investigation.

This study has several limitations. First, as a cross-sectional analysis, our findings can only establish associations, not causal relationships, between ePWV and CKD. Longitudinal studies are needed to explore the role of ePWV in CKD onset and progression. For instance, a recent study has revealed that vascular and metabolic dysfunctions accumulate gradually over time even in genetically predisposed individuals (38), supporting the necessity of longitudinal assessments in investigating the relationship between ePWV and CKD. Second, NHANES data may not capture all potential confounders, and reliance on self-reported data introduces the risk of residual confounding. Third, our findings are based on a U. S. population and may not be generalizable to other regions or populations with differing socioeconomic or racial/ethnic characteristics. The strong association between APOL1 risk variants and renal damage in populations of African ancestry, along with the specific eQTL signals of the NFATC1 pathway in individuals of African or Americas ancestry, suggests that genetic variations across different ethnic groups may synergistically influence renal function and vascular compliance through unique molecular mechanisms, including salt sensitivity and immune-inflammatory responses (39). Consequently, this can lead to population-specific differences in the association between ePWV and CKD. Finally, there are limitations to using ePWV as a surrogate for PWV, as it is estimated solely based on age and mean arterial pressure. This approach may overlook subtle vascular alterations and fail to adequately reflect the impact of immune status on blood vessels (40), potentially hindering early disease diagnosis.

Future research should focus on the longitudinal relationship between ePWV and CKD, accounting for time-varying factors. Simultaneously, further prospective studies are necessary to develop and validate a multi-parameter risk prediction model that integrates assessments of vascular function, metabolism, and inflammation. Such a model will enhance the predictive power for cardiovascular events and renal outcomes in patients with CKD. Additionally, studies in diverse populations could enhance the generalizability of our findings and further validate the utility of ePWV in assessing vascular and renal health. Finally, given the complexity of the ePWV calculation equation, our research team intends to develop a web-based ePWV calculator in HTML format, or a WeChat mini program, to facilitate its use by primary care practitioners.

## 5 Conclusion

In general, this study demonstrates a strong positive association between ePWV and CKD prevalence, indicating the potential utility of ePWV monitoring in predicting CKD prevalence. Prospective investigations are essential to substantiate these findings and to ascertain the presence of causal links.

## Data Availability

The original contributions presented in the study are included in the article/supplementary material, further inquiries can be directed to the corresponding author.

## Footnotes

^1^ www.cdc.gov/nchs/nhanes/
